# Use of the Dynamic Systems Development Method to Inform Technology-Assisted Motivational Interviewing (TAMI) for Tobacco Cessation: Qualitative Study

**DOI:** 10.2196/88125

**Published:** 2026-06-02

**Authors:** Brian Borsari, Joannalyn Delacruz, Ahson Saiyed, John Layton, Karla D Llanes, Isaac A Mirzadegan, Jing Cheng, Anita S Hargrave-Bouagnon, Meredith C Meacham, Delwyn Catley, Jason Satterfield

**Affiliations:** 1 San Francisco VA Medical Center, 4150 Clement St (116B), San Francisco, CA, 94121, United States, 1 415-221-4810 ext 26078; 2 Department of Psychiatry and Behavioral Sciences, University of California, San Francisco, San Francisco, CA, United States; 3 Open Health Network, Arlington, VA, United States; 4 Pharmaceutical Sciences, School of Pharmacy, The University of Texas at El Paso, El Paso, TX, United States; 5 School of Dentistry, University of California, San Francisco, San Francisco, CA, United States; 6 School of Medicine, University of California, San Francisco, San Francisco, CA, United States; 7 Department of Psychology, San Diego State University, San Diego, CA, United States

**Keywords:** mHealth, nicotine, motivational interviewing, chatbot, qualitative, machine learning, mobile health

## Abstract

**Background:**

Smoking continues to be a leading cause of preventable morbidity and mortality, and more than 480,000 Americans die annually due to smoking-related illness attributable to smoking and secondhand smoke. More advanced, responsive, and tailored digital interventions using machine learning and artificial intelligence may be a valuable tool for successful smoking cessation referrals.

**Objective:**

This study used the dynamic systems development method to incorporate patient and consumer sources of conversational data to develop a technology-assisted motivational interviewing (TAMI) chatbot, a digital agent using machine learning models to deliver motivational interviewing (MI) for tobacco cessation.

**Methods:**

During the functional model iteration phase, user-centered design interviews with smokers (n=3) informed the creation of TAMI. The design and build phase involved the use of existing datasets to guide the incorporation of MI-consistent utterances, language recognition, and topic classification to guide a discussion about smoking, and providing a tailored quit plan if indicated. During the implementation phase, user experience interviews with randomly selected participants (n=9) in a pilot trial discussed their experiences with TAMI.

**Results:**

User-centered design interviews indicated a desire for a chatbot that was engaging and adaptable to personal interests in quitting smoking. Inductive analysis of user experience interviews revealed that anonymity, regular reminders, and a humanized experience facilitated engagement with TAMI, but technical glitches, chatbot misunderstandings, and issues with rapport were barriers to engagement.

**Conclusions:**

Informed by user input and patient and consumer datasets, TAMI can use MI skills to elicit change talk and/or accurately evaluate readiness for tobacco cessation. Further development will enhance TAMI’s ability to seamlessly engage with users when discussing behavior change and assist underserved populations achieve improvements in a variety of health behavior goals.

## Introduction

### Health Risks Related to Smoking

Smoking continues to be a leading cause of preventable morbidity and mortality. An estimated 11.5% of American adults currently smoke [1], and more than 480,000 die annually due to smoking-related illness attributable to smoking and secondhand smoke [2,3]. Individuals with low education, low income, and those from marginalized minority groups or residing in rural areas are disproportionately affected [4].

### The Use of Digital Health and Chatbots to Address Smoking

Smartphones have extensive reach into even low-income populations and are increasingly integrated into traditional health care [5,6]. Although smartphone-based or other digital interventions for smoking cessation are not new, the vast majority are unvalidated mobile apps that typically require motivated users and rarely engage patients in a sustained and meaningful way [7]. More advanced, responsive, and tailored digital interventions using machine learning and artificial intelligence (AI)—such as chatbots that empower patients and support providers—are necessary to harness the true promise of digital health as a tool for active (and successful) cessation referrals [8-10]. Chatbot research has shown that patients enjoy interacting with chatbots several times per day [11], develop emotional attachments [12], and show high levels of satisfaction [13]—even preferring the chatbot to a human [14,15]. However, like other digital health tools for smoking cessation, the chatbot needs to be adapted to fit individuals’ needs. This includes recognizing users’ diverse technological skills, accommodating busy schedules, addressing privacy concerns about data storage and use, reducing overreliance on chatbots for health care needs, and increasing engagement [16-19]. Despite concerns regarding the accuracy and safety of health-related AI chatbots, studies largely show that they are acceptable and can be beneficial to patients [20]. Even if a digital tool has initial appeal, extended patient engagement is unlikely [16,17]. Therefore, the style of communication any chatbot uses to discuss smoking is a vital consideration.

### Motivational Interviewing

Motivational interviewing (MI) is “a particular way of talking to people about change and growth to strengthen their own motivation and commitment” [21; pp. 12‐13]. In several reviews and meta-analyses, MI has been observed as an efficacious intervention for substance abuse behaviors, with small to moderate and variable effects [22-27]. Unlike other digital health tools that rely on didactic content, MI is a collaborative communication style that elicits and strengthens personal motivation for change through open-ended dialogue and reflections. MI may be an appropriate communication style to enhance one’s intrinsic interest in a referral to quit smoking. Not a technique or tool, MI is a therapeutic conversational approach to elicit change talk in support of behavior change. Change talk (CT) includes statements that “indicate movement toward a particular change”; in contrast, sustain talk (ST) “moves the speaker away from change in support of the status quo” [28; p. 25]. Current MI theory [21,28] posits that increasing CT and reducing ST increases the likelihood that individuals will change subsequent behavior. Indeed, a greater proportion of CT is significantly associated with a reduction in risk behaviors [29]. Two therapeutic components facilitate this task: a relational component and a technical component [30,31]. The relational component focuses on the clinical relationship that develops between the therapist and the client over the course of the session (eg, therapist empathy). The technical component comprises specific MI-consistent (MICO) therapist skills (eg, reflections, affirmations, and open-ended questions about the benefits of change) associated with increased CT and decreased ST, which in turn should lead to desired health behavior outcomes. Meta-analyses of in-session processes of MI indicate the most support for the technical component (which can be readily translated into a chatbot) in predicting behavior [32], a finding replicated in subsequent meta-analyses [24,29].

### Limitations of Mobile Health and Chatbots Incorporating Motivational Interviewing

Digital tools incorporating MICO skills have been developed for a variety of health behaviors [33,34]. A 2016 review of 41 studies [35] examining technology-delivered adaptation of MI found that the majority (32/41) delivered fully automated interventions with no interaction with counselors, and only 5 used text interactions with counselors using the technology (eg, chat room and computer program) in real time. Since that review, there have been several more advanced, responsive, and tailored digital interventions—such as chatbots that empower patients and support providers—that try to harness the true promise of digital health as a tool for active (and successful) cessation referrals [8,36,37]. Thus, advances in machine learning and AI can improve the delivery of MICO technical skills, thus enhancing the focus and credibility of the MI delivered and enhancing the relational components of MI (empathy, collaboration, and facilitating self-exploration). For example, one study found that a smoking cessation MI chatbot was able to increase self-efficacy by simply programming the MI chatbot to ask open-ended evocative questions; similarly, programming reflections using ChatGPT in addition to evocative questions improved readiness to quit and perceived empathy of the MI chatbot [34]. However, these interactions were often brief, invariably structured, and followed a regular sequence (eg, question, reflection, and question). Therefore, it remains unknown how well a chatbot can have a longer iterative conversation about different topics associated with smoking.

### This Study

This study details how a technology-assisted motivational interviewing (TAMI) chatbot was developed. The process drew on agile methodology—the dynamic systems development method (DSDM) [38,39]—which is an incremental approach that can incorporate client feedback at different phases of chatbot development. We detail our efforts in 3 phases of the DSDM: the functional model iteration phase, the design and build phase, and the implementation phase. During these phases, we incorporated user-centered design interviews, existing patient and consumer datasets, and user experience interviews to ensure the meaningful involvement of patients with lived experience in the design, development, and deployment of an AI application for smoking cessation. The goal of our work was to develop a chatbot that could evoke and resolve ambivalence about quitting smoking and provide a personalized quit plan to accomplish this goal.

## Methods

### Study Design

The DSDM consists of 5 phases (see Textbox 1). During the first 2 phases, the feasibility study phase and the business study phase, the research team specified that a credible chatbot will ideally engage the user, maintain a conversational focus on smoking to enhance motivation to quit smoking, and then develop a personalized quit plan. As part of the functional model iteration phase, the concept of a chatbot was then presented to 3 current smokers for evaluation in order to identify which aspects of the chatbot were worth further development.

Textbox 1. **Dynamic systems development method (DSDM) phases.**

1. Feasibility study phase
  - Determine the feasibility of the project given the personnel, funding, and timeline
  - Determine technical aspects of technology-assisted motivational interviewing (TAMI) chatbot that will be appropriate (eg, format and server location)
2. Business study phase
  - Identify key functionalities of TAMI (eg, text prompts and quit plan)
  - Outline TAMI prototype
3. Functional model iteration phase
  - Build an initial scenario, identify an initial set of intents (topics), and responses
  - User-centered design interviews (n=3) presenting the initial prototype of TAMI
4. Design and build phase
  - Use a library of existing texts (eg, motivational interviewing [MI] transcripts) and extend existing statistical models (eg, Word2Vec) to build and test an intent recognition model
  - Build a statistical model recognizing and labeling the change in language and the degree of its use
  - Implement initial conversation flow using a decision tree and a database of potential intents; train a statistical decision tree model using existing conversation flows
  - Run initial user tests by project staff, with the goal of “tripping up” the system and finding misunderstood intents and badly worded responses
  - Refine the statistical models and repeat steps 1-2 until users complete the entire conversation with TAMI in 85% or more of cases
5. Implementation phase
  - Adult smokers recruited from primary care clinics were randomized to receive TAMI (n=39) or the usual-care control condition (n=421)
  - TAMI participants:
    - were instructed to have ≥12 unsupervised sessions over the course of the 6-month study, and also to go at their own pace and to use the web application whenever desired
    - set the frequency with which they would receive text reminders to use TAMI (twice a week, weekly, or every other week)
  - User experience interviews were conducted with a randomly selected subset of patients who used TAMI (n=9)

### Ethics Approval

All procedures received approval from the University of California, San Francisco institutional review board (#19‐28845).

### User-Centered Design Interviews: DSDM Step 3 of 5

Three participants (2 female and 1 male; 100% White) were recruited from a primary care clinic via posted fliers. They were English-speaking, owned a smartphone, and reported smoking at least 100 cigarettes in their lifetime and smoked at least one cigarette per day for the past 7 days. Previous experience with chatbots was not a requirement for participation. Eligible patients who did not opt out of screening were contacted by the study coordinator, and those who agreed to participate were scheduled for a study onboarding session conducted via Zoom by the study coordinator. At onboarding, the study coordinator reviewed the consent form with each patient, obtaining informed consent via the ask-teach-ask method. Upon obtaining consent, 30‐45 minute interviews were conducted via Zoom by the study coordinator. Participants were reminded that responses were confidential and all feedback (ie, positive and negative) was welcomed to improve the TAMI chatbot. Participants received an electronic US $30 gift card. Participants were told, “Today we are discussing the design of a chatbot that helps patients get ready for smoking cessation referrals. We have done the initial programming and have some design ideas but we want your input before we get too far along.” The participants were then asked (1) how they would feel about interacting with TAMI, (2) the best way for TAMI to ask if someone is ready to quit smoking, and (3) how TAMI could help someone in creating a quit plan.

## Results

### User-Centered Design Interviews: Qualitative Analysis

The 3 interviews were transcribed by Otter.ai voice-to-text software. After transcripts were transcribed and cleaned by listening to audio and comparing the transcribed text, we used a grounded theory inductive approach [40] to understand individuals’ recommendations about TAMI. Two qualitative experts of the study team read the transcribed interviews to generate summary notes to become familiar with the interviews. The summary notes were then used to identify the themes to create a consensus codebook. The identified themes were grouped based on the 3 research questions.

*RQ1: How would participants feel about interacting with TAMI?*

Participants wanted a sense of connection with TAMI in two ways. First, they wanted TAMI to be able to smoothly interact with them by picking up social cues (eg, using and responding to humor and sarcasm) and not have sterile or rudimentary responses. Second, there was a desire to feel understood as a smoker, and to have their personal experience of smoking and motivation to quit evoked and affirmed.

*[When communicating with a chatbot,] I feel like I’m putting in as much work trying to get the bot to understand what I’m trying to go for...it’s not as helpful as I would find it. I feel like I’m trying to help* it *[to] help* me.

[Participant 1]

*RQ2: What is the best way for TAMI to ask if someone is ready to quit smoking?*

Participants expressed that TAMI should understand the balance between checking in on their motivation to quit smoking versus being too “pushy.” Furthermore, information provided should be sensitive to the content of the check-ins, rather than standard or boilerplate educational materials about the benefits of quitting. Interactions should be friendly, inquisitive, and encouraging, rather than lecturing, advice-giving, or shaming. Ideally, check-ins would occur less frequently (eg, weekly and monthly) when the individual is considering quitting or the quit date is far away.

*Continue to check in once a month. You know, not, not any more frequently than that, but still check in, you know, to make sure…and to kind of bolster that person’s confidence that somebody’s thinking about them*.

[Participant 2]

Then, interactions would ramp up in frequency as the user approaches a quit date and reach their highest frequency during the acute cessation period. Across multiple chat sessions, TAMI should recall previous discussions to avoid participants having to repeat themselves.

*RQ3: How can TAMI help someone in creating a quit plan?*

Participants wanted a tailored menu of community options and strategies that they could then select and pursue, rather than having a prescriptive set of instructions. Options provided should reflect users’ ideas about optimal ways to quit (eg, nicotine replacement therapy and nonmedication options such as stress-management groups), and interviewees also expressed a desire for a menu of strategies they could use to weather urges to smoke. Information about the immediate and ongoing benefits of quitting (eg, positive effects on the lungs and body, financial savings for a desired item) was also seen as valuable to enhance motivation to quit. Participants wanted to easily access in-depth information about various options, but with only the key points displayed on the front page of the quit plan. Emailing or texting the formal quit plan was acceptable, and all participants agreed that the quit date should be prominently displayed. Participants recommended flexibility in changing the quit plan if needed, as well as having input on when TAMI would contact them.

Taken together, the user-centered design interviews confirmed that MI would be an ideal approach for the TAMI chatbot for several reasons. First, MI has 4 tasks that are sequentially addressed during an MICO conversation about change: engaging the client, focusing on a specific behavior, evoking a person’s own argument in favor of change, and planning behavior change. Second, there are specific MICO skills that the chatbot can use (eg, open-ended questions) to evoke information from users about their smoking, which can then be reflected to foster a user’s sense of being understood. Third, evoked CT and ST can indicate the user’s motivation and interest in changing smoking. Finally, MI is an eliciting approach, rather than a confrontational or prescriptive style of communication.

### Design and Build Phase: DSDM Step 4 of 5

#### TAMI Prototype

The DSDM design and build phase then commenced and included the development of a prototype of TAMI designed to guide the user through the 4 tasks of MI. This process relied on data from existing patients (eg, MI transcripts) and consumers (eg, Facebook posts and public Reddit threads). Extending existing statistical models (eg, Word2Vec), an intent recognition model was built and tested. The initial construction of TAMI was based on over 450 session transcripts, training codebooks, role-play transcripts, and expert input to map basic conversational structures and develop a lexicon of CT with linked decision trees driving TAMI’s MICO responses. Table 1 provides an overview and summary of patient and consumer data [41] used to inform both the focusing and evoking components of TAMI. In addition, during the design and build phase, there were multiple iterative development rounds with project staff, with the goal of “tripping up” the system and finding misunderstood intents and badly worded responses, which taught the chatbot to be more intelligent and MI-consistent in its responses.

**Table 1.** Data sources used in the development of TAMI^a^.

Data source	Description	Data type	Sample description	Task/data extraction
Meacham et al [42]	Short textual language about smoking	Consumer	5416 utterances classified for topic by the clinical research coding team	Evocation: change talk/sustain talk identification
Catley et al [43]	Motivational interviewing and health education for smoking	Patient	Over 10,000 utterances classified by the clinical research coding team	Focusing: topic classification Evocation: change talk identification
Reddit	Discussions about 15 smoking topics	Consumer	17,225 utterances	Focusing: topic classification
Borsari et al [44]	Motivational interviewing for alcohol use	Patient	46,301 utterances parsed by machine and change talk identification by the clinical research coding team	Evocation: change talk identification
Apodaca et al [45]	Motivational interviewing for alcohol use	Patient	Sessions reviewed for session theme development	Evocation: change talk identification

a TAMI: technology-assisted motivational interviewing.

#### Development of the Engaging Task

One of the challenges of any digital tool is facilitating repeated engagement, which has also been termed a working relationship, connection, or alliance [21]. Therefore, TAMI reaches out to users on a schedule tailored to their preferences. As a result, users need not remember to open an application or do their “homework,” as the chatbot will prompt them at optimal (predetermined) time points throughout the week, engage them in brief conversations, and (periodically) recommend activities or other homework that is later reviewed in future conversations. Although a minimum number of contacts can be programmed to occur, users were also able to initiate conversations as often as they liked and at any time. Moreover, users were able to determine what times they preferred being contacted. The default frequency was once a week, although this could be increased or decreased by the user. In addition, if a user had decided to quit and received a referral, notifications from TAMI ceased. Together, these efforts ensured that TAMI engaged the users at appropriate times to discuss smoking.

#### Development of the Focusing Task

Once the user was engaged, TAMI needed to maintain a clear and consistent conversational direction about change toward smoking cessation rather than being sidetracked to other topics. Focusing was conceptualized as having two components: (1) structured, discrete sessions in which the user could discuss their smoking and (2) the prompting of specific topics regarding smoking.

##### Session Structure

As TAMI responded to only the previous user text, keeping the focus of smoking could be quickly lost. To this end, session templates were constructed for each of 12 themed sessions that explored topics such as the pros and cons of smoking, benefits of quitting, self-efficacy, and setting a quit date (see Table 1). MI theory informed the development of the structure of sessions—specifically the use of MICO skills of open-ended questions, affirmations, and reflections to guide the user through the session. In addition, TAMI incorporated MI strategies such as the readiness ruler and asking key questions (“What do you think you’ll decide to do?”).

##### Smoking Topics

To identify topics that were relevant to smokers, we examined deidentified transcripts from a clinical trial randomized smokers to receive in-person sessions providing health education or MI [43] as well as chats from a trial delivering a smoking intervention via Facebook [42]. These data sources yielded 81 topics related to smoking, such as financial costs of smoking, appearance (breath, smell), social triggers (family, friends), health care systems, and mental health. These topics were then entered into Reddit, an online forum of conversations about a wide variety of topics, in order to extract posts and comments that incorporated those topics. These discussions were reviewed and off-topic information was removed. This dataset was then divided into 77 classes to enhance topic comprehension and guide TAMI’s response to the user.

### Development of the Evocation Task

Once the user was engaged and focused on the session, the challenge was to have TAMI be able to classify the user texts (by topic, CT, and ST) and then respond appropriately using MICO skills that have been demonstrated to evoke further (eg, double-sided reflections ending on user CT) [28].

#### Testing Datasets

A large part of TAMI’s design with respect to reflection and language-recognition sought to identify topics and CT from existing clinical MI datasets and infuse these with AI engagement. An existing corpus of MI transcripts was used to map conversational structures and derive machine learning–based classifiers allowing recognition of message intents, client change-language content, and decision trees for accurate MICO responses. Datasets were machine-parsed into utterances, and then the clinical research coding team assessed the presence of CT within the utterances and identified topic and subtopic classification. Two datasets [44,45] had already reliably coded client language, and the clinical research coding team identified CT and ST within the other datasets by consensus. TAMI was then able to incorporate data that had already been parsed (divided into discrete utterances) and coded (see Table 2). However, other data that the team accessed had to be put into a format that TAMI could incorporate, such as more than 20,000 lines of deidentified utterances from the Facebook dataset and a prior randomized controlled trial with unmotivated community member smokers [43]. This took 3 steps. First, the data were transcribed from audio to written text. For all received written transcripts, this process was done manually; for audio files, Otter.ai was used. Second, the data were parsed into utterances using natural language processing (NLP). Third, the utterances were coded into CT and ST by a group of coders trained by the research team [46].

**Table 2.** Motivational interviewing session template themes.

Session	Session themes
1	Rapport-building, exploring the pros and cons of smoking, and assessment of readiness using the readiness ladder
2	How quitting smoking would affect your life
3	Tackling a problem related to quitting smoking
4	Discussing and celebrating previous quit attempts
5	Overview of facts related to smoking and smoking cessation
6	Exploring the risks and rewards of smoking
7	Reviewing evidence-based smoking cessation interventions
8	Anticipating obstacles that could get in the way of quitting smoking
9	Imagining life as a nonsmoker
10	Reviewing pharmacologic smoking cessation tools
11	How would you advise a friend or family member who wants to quit smoking?
12	Reflecting on the past 3 months of the intervention, giving information about how to make a quit plan on the TAMI^a^ Coach web application

a TAMI: technology-assisted motivational interviewing.

TAMI’s custom Natural Language Understanding model–based approach evaluated utterances using transformer-based classifiers. In addition to topic and CT/ST classification, MICO skills and question-answer classification were also added [46]. Using these scored and coded transcripts, the team trained a classifier for each situation. An existing neural network, Word2Vec, was used [47] that had been pretrained on the English language corpus derived from Wikipedia articles. Word2Vec represents synonyms and antonyms in the English language without explicit hand-built dictionaries. Word2Vec measures how similar the user-entered text is to sets of texts representing specific topics (eg, smoking and shortness of breath). A piece of text that meets a certain threshold is seen as expressing that “specific intent.” Having a library of “intents” and associated training text thus permitted the refinement and filtering of the output of a neural network and use it to generate accurate responses. The primary challenge of this phase was managing the breadth of potential user responses, categorizing them correctly, then having TAMI recognize the “topic intent” of the user and respond with MICO skills [46].

#### Response Libraries

Once TAMI was able to classify the text from the user, a MICO response had to be generated. Therefore, a library of reflections and open-ended questions was developed to ensure that the conversational abilities of TAMI were always MI-Consistent (see Multimedia Appendix 1). Affirmations recognizing effort and engagement with TAMI were generated and delivered at certain points during the MI modules. In contrast, reflections were organized into a 77-class-by-5-decision (strong and moderate CT, neutral, strong and moderate ST) matrix, resulting in 385 possible responses, which would enhance accuracy and avoid redundant responses. The matrix was reviewed and edited by clinicians and expert coders. The same approach was used to develop a matrix for open-ended questions.

#### Identification of Change Talk

**Figure 1.**
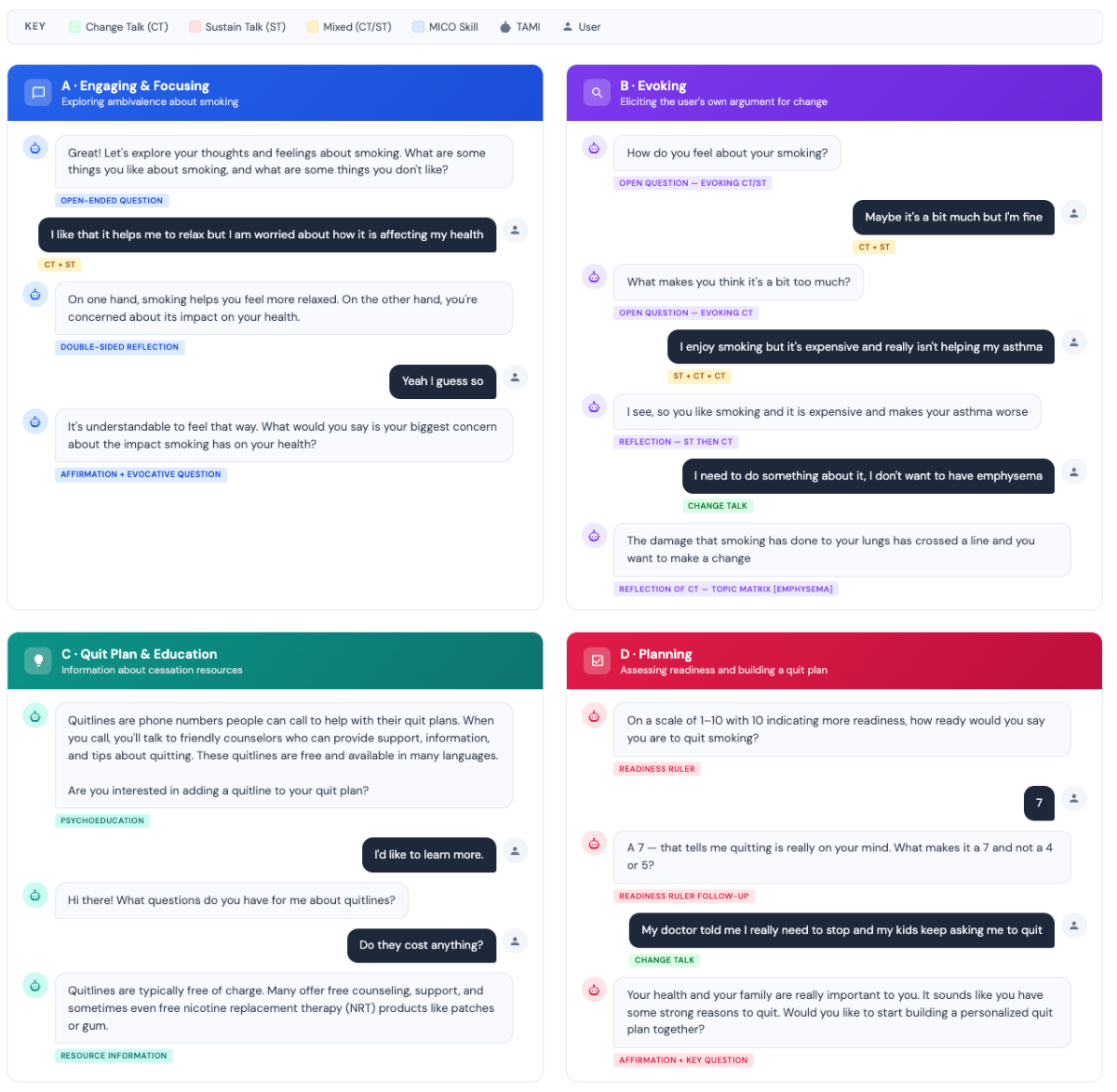
Screenshots of TAMI (technology-assisted motivational interviewing chatbot).

TAMI was trained to identify CT and ST for tobacco use using a neural network, BERT (Bidirectional Encoder Representations From Transformers) [48], to build a model of CT versus ST in an MI interview context. We trained the neural network on 51,000 utterances extracted from 255 deidentified transcripts [44,45]. BERT network recognized CT/ST with an *F*_1_-score of 0.88—comparable accuracy to a human coder. As seen in the interaction detailed in Figure 1, these efforts facilitated the incorporation of MICO prompts to enhance relational connection to TAMI and elicit CT.

#### Development of Decision Tree and Matrix

After the development of an NLP model, TAMI chat flow diagrams were developed, and the response matrix and decision tree were reviewed. A demo reviewed utterance input and return vectors for CT and ST, as well as topic classification. The connection of these vectors to the matrices of responses led to the next step of TAMI development, addressing issues such as repeated questions by a user.

### Development of the Planning Task

Once a user completed a session with TAMI and indicated readiness to quit smoking, TAMI guided them through personal preferences for referral and quit-plan elements similar to our prior work [49,50] and suggestions from the user design interviews (eg, a menu of external resources).

The tailored quit plan was typically comprised of several evidence-based cessation interventions that are selected by the user, such as nicotine replacement therapy, behavioral counseling, state telephonic quitlines, and pharmacotherapy (eg, varenicline). TAMI evoked strategies used in the past and reminded users when additional steps need to be taken, such as contacting a prescribing physician or downloading discount coupons to buy nicotine patches. TAMI also encouraged the user to set a quit date and provided tips to increase the likelihood of success, such as removing smoking triggers from the home and informing friends and family about the quit date. The full, tailored quit plan with the quit date prominently displayed as well as relevant links and instructions were downloadable and printable.

### Implementation Phase: DSDM Step 5 of 5

#### TAMI Implementation

TAMI was implemented with adult smokers recruited from primary care clinics for a feasibility and acceptability pilot. Inclusion criteria were (1) age of 18 years or older, (2) ability to speak and read English, (3) ownership of a smartphone, (4) daily smoker, smoking one or more cigarettes per day in the past 7 days, and (5) smoking at least 100 cigarettes in their lifetime. Participants were randomized to receive either TAMI (n=39) or the usual-care control condition (n=41). Participants in the usual care condition were provided an educational handout and suggestions for local smoking-cessation referrals.

Participants assigned to TAMI were given a URL to TAMI and asked to complete their first session with TAMI. The study coordinator was immediately available to answer any questions or address any barriers to accessing the web application. Participants were instructed to have at least 12 unsupervised sessions with TAMI over the course of the 6-month study. Each session varied in duration, ranging from 5 to 15 minutes. Participants set the frequency with which they would receive SMS text reminders to use the TAMI Coach (twice a week, weekly, or every other week). However, participants were also encouraged to go at their own pace and use the web application whenever they desired.

Any participant who completed a quit plan and received a tailored referral from the TAMI Coach was instructed to stop using the TAMI Coach and seek out the resources listed on their referral document. For these participants, SMS text reminders no longer encouraged engagement with the TAMI Coach web application; instead, the reminders offered words of support and encouraged engagement with the tailored referral resources.

#### User Experience Interviews

To develop a deeper understanding of an individual’s experience interacting with TAMI, recruitment from the TAMI group was random and stratified to include participants who set a quit plan, and those who did not. Nine female participants (44% White) agreed to be interviewed in exchange for a US $50 electronic gift card. These interviews ranged from 20 to 30 minutes and were conducted via Zoom videoconferencing platform by the study coordinator. Participants were reminded that responses were confidential and all feedback (ie, positive and negative) was welcomed to improve TAMI. Questions were asked about what participants liked/what was most helpful about TAMI, what participants disliked/what was least helpful about TAMI, and how TAMI could be improved to help people quit smoking.

### User Experience Interviews: Qualitative Analysis

Interviews were transcribed, cleaned, and uploaded to Atlas.ti to assist with data management and inputting the codebook. We used a grounded theory inductive approach [40] to understand individuals’ experiences with TAMI. One qualitative expert from the study team read all transcribed interviews to generate summary notes to become familiar with the interviews. The summary notes were then used to identify themes to create a codebook. The identified themes were grouped based on the 3 research questions.

*RQ1: What did participants like/what was most helpful about TAMI?*

Participants reported liking TAMI for various reasons: TAMI was readily available on their phones, it provided resources to help quit smoking if they were interested in quitting, and TAMI was capable of asking a variety of questions. Response to the check-ins was positive, and one participant felt it humanized the experience:

*It was cool because it was random…And it just reach[ed] me at a good time where I had a minute to, you know, respond. I was like, “Oh, well, I just quit smoking”...It was able to humanize the experience a little more*.

[Participant 76]

Others felt that the reminder check-in showed TAMI cared:

*I would say [what] was good about her, too, is that, like, the tone of the messages was like, questioning but not in a pushy way. And it wasn’t like didactic…I think if it had been like stats about how bad smoking is for you, I would have hung up*.

[Participant 90]

Digital interventions provide users with some anonymity. TAMI provided P48 with a private workspace to feel comfortable working through her quitting process and not be judged, versus doing it with other people like friends, family, or a therapist who might pressure her too much:

*It’s at the end, like it was only me, and you know, this. You call it AI? Yeah. Right. That doesn’t know me, does not care. You know, [doesn’t care] like, what I do, but it’s asking these questions. And it’s up to me if I want to follow through with what I’m saying or not*.

[Participant 48]

*RQ2: What was disliked/least helpful about TAMI?*

Some participants felt TAMI being nonhuman made it difficult to express themself emotionally, but the main issue participants reported was that TAMI did not understand or respond appropriately in some conversations:

*You know, as like, [TAMI is] not remembering what I said…you have no clue [that] you’re not responding to my questions and/or my statements or responding appropriately and all of that sort of stuff*.

[Participant 75]

In addition, personal preferences in who to communicate with and the impersonal nature of TAMI appeared to affect motivation to connect and share personal information about smoking.

*It’s also just my personality, I’m not necessarily just going to bare my soul via text. Unless it’s to a person I really know. Perhaps other people are fine with like an anonymous TAMI, and all of that. But for me, and especially if the response I receive to my comments or questions don’t have a bearing on what I asked or said, then it’s kind of like, why should I even bother to write, to sit here and write.*

[Participant 75]

Participants who did not express interest in quitting and therefore did not receive a quit plan expressed that they still would have liked to be given options to help them quit. Some participants who did receive a quit plan felt some of the suggestions would take too long.

*RQ3: How can TAMI be improved to help people quit smoking?*

Participants offered several suggestions to improve TAMI. Other participants thought TAMI could increase engagement by remembering names or previous conversations. Participants adopted strategies to help them quit after setting a quit date. One individual felt that a tracking component would help individuals understand their smoking better; others recommended providing information about cessation options before formally establishing a quit plan.

*Sometimes people might not think they’re ready. And when they find something that interests them and they’re like, “oh, that might work for me.” And then it gets a thought in their head. And they think about it. And then they’re like, “Okay, this, I’m going to do this.” And that might be a better avenue than, like, “let’s create a plan.”*

[Participant 103]

Using TAMI to connect with others was also mentioned by participants. One suggestion was a peer support group or chat option to see other people’s struggles, not feel so alone, and learn what has been helpful to others when quitting.

*I know, [with] quitting, you feel alone, you feel alone. And I constantly I would look at other people and be like, “Wow, they’re so lucky that this isn’t like weighing down on them.” And I’m sure that’s true…I mean, if ,yeah, there’s other people around or, you know, positive messages from people*…

[Participant 48]

One participant stated it would be helpful to speak to a human health coach with more knowledge for approximately 20-minute sessions every week after setting a quit date. Other participants wanted features to make the experience more personal, such as an Avatar that looked like them. Another individual wanted TAMI to develop a deeper connection:

*Something like, you know, rather than just a nebulous, “how can I help?” or “sorry to hear that” or something. So, to really kind of make a connection to the person that TAMI is trying to help. Now, I don’t know exactly if that’s possible with AI and TAMI.*

[Participant 75]

## Discussion

### Overview

These findings demonstrate the value of using agile methodology to incorporate user-centered feedback and data across multiple development phases, particularly in aligning chatbot tone and content with MI principles. Through the involvement and consultation of patients who were considering quitting smoking, as well as using patient and consumer datasets, the final iteration of TAMI was a fully automated chatbot that uses NLP to “understand” what a patient is saying, followed by the selection of an appropriately matched, prewritten response. Every time a user initiates a conversation with TAMI via a secure website, it assesses readiness to quit smoking, and—depending on the user’s stage of change—TAMI will either begin a “module” of MI to enhance readiness or TAMI guides the user in the creation of a tailored quit plan to quit smoking in the next 30 days. This structured, button-driven module “tours” the user through several evidence-based smoking cessation interventions and asks the user to choose their preferred “quit tool(s).” After the quit tools have been selected, the TAMI Coach asks the user to set a quit date and provides advice regarding quit-day preparations and coping with cravings. A final PDF quit-plan summary is produced that includes instructions on how/when to access the quit tools including community referrals, and access to resources such as low-cost nicotine replacement therapy or local quitlines. By combining proven engagement strategies (eg, targeted incentives, an active chatbot vs passive mobile app) with AI and machine learning, TAMI helps to enhance patient motivation and reduce barriers to utilization by tailoring referrals that are provided at just the right time for all patients, regardless of their initial level of readiness. Our use of the DSDM provided several key findings, and limitations of the current project suggest promising future areas of research.

### Key Findings

First and foremost, MI was the appropriate approach to use with this population as user-centered design interviews highlighted a desire to feel understood, affirmed, and not judged. Furthermore, MI was consistent with the request for evocation and support for personal motivation to quit, which in turn could inform the balance between providing relevant advice and suggestions and giving information to inform but not dictate decisions. As such, TAMI was designed to use compelling and high-fidelity MICO skills such as affirmation, and reflections to guide the patient through the 4 tasks of MI—engagement, a focus on smoking, evoking and resolving ambivalence about quitting smoking, and planning for change. Consistent with MI, the incorporation of MICO skills may also enhance the relational components of MI: Participants felt that TAMI could express empathy by providing a variety of responses and unintrusive check-ins and open-ended questions. The themes identified about what was liked about TAMI are consistent with the MI spirit [28]: The participant felt cared for or that TAMI humanized the experience is important when delivering MI. The judgment-free anonymity component of TAMI is consistent with MI principles of conveying empathy and exploring both the cons and benefits of quitting without judgment.

Other clear preferences emerged during our work with patients who were considering quitting smoking. First, the frequency of the check-ins was linked to the patient’s readiness to quit smoking. Participants expressed that they appreciated how TAMI regularly checked in on their progress and, despite being from a chatbot, these check-ins made them feel cared for during their quitting journey. As with all interventions, the timing of the delivery of TAMI is also important, as frequent check-ins if one is not considering quitting smoking were perceived as potentially annoying. In contrast, frequent contact leading up to and following the quit date was seen as helpful to keep the user focused. Second, there was also a desire to be provided with a variety of tips and external resources to facilitate quitting, thus enabling the user to select which would be most helpful. Inductive analysis of exit interviews revealed that anonymity, regular reminders, and a humanized experience facilitated engagement with TAMI. TAMI does not require any provider involvement, although it allows for and encourages exploring professional assistance with quitting smoking when developing a quit plan. Finally, participants may like the anonymity digital interventions provide when trying to quit smoking, particularly the opportunity to privately explore ambivalence about smoking and options for cessation.

### Limitations and Future Directions

Limitations to this study indicate several aspects of TAMI that require improvement. First, while the anonymity component was liked by some participants, one participant expressed difficulty connecting with the chatbot emotionally, which could be a limitation of the relational component of using TAMI. Second, some individuals felt TAMI did not understand them or their responses. The occasional glitches may leave a person not feeling understood. Thus, TAMI needs to be capable of providing complex reflections that convey an accurate understanding of the user’s personal experiences with smoking. Refinement of the reflections is needed to improve the technical component and strengthen the relational component of TAMI. The technical glitches and relational limitations may be barriers to sustained engagement. Third, as with all interventions, the timing of the delivery of TAMI is also important, as some individuals were not ready to quit smoking and reported that was the reason they did not feel the need for TAMI. Finally, there was limited diversity in those who completed the interviews, especially in the user design interview phase. Therefore, it will be vital to recruit a more diverse group of participants in the subsequent development and testing of TAMI.

A key challenge was balancing structured content delivery with the flexibility needed to simulate a natural, responsive dialogue—a limitation that highlights the need for more dynamic conversational AI. TAMI remains a largely scripted and structured intervention with limited ability to adapt to participant input and questions, thus potentially limiting engagement and follow-through with the quit plan. Additional data regarding pilot feasibility-and-acceptability outcomes will be reported in a future paper. Fortunately, since this work was completed, there have been remarkable advances in the development of large language models (eg, ChatGPT 4.0) that incorporate large amounts of data to craft questions and reflections that are equivalent to human-generated utterances in criteria such as specificity, naturalness, appropriateness, and engagement [34,51,52]. For example, Therabot is a chatbot for mental health treatment that was recently deployed among individuals reporting symptoms of depression and anxiety [53] and was capable of several conversations per day over 8 weeks. Previous conversation content was incorporated into Therabot prompts that included empathic responses, questions, or specific relevant information. However, all prompts were monitored by members of the research team who contacted participants to correct responses (13 times over the course of the study) or further assessment for risk (eg, suicidality; 15 times). As a result, the next steps for the further development of TAMI are to effectively graft the structure of TAMI with more advanced large language models to create opportunities for enhanced evocations and strengthening of the participant’s personal argument for quitting smoking without monitoring. This enhancement will be especially important during the evocation component, which was previously limited to scripted reflections and questions pertinent only to the most immediate response by the participant. Specifically, the chatbot may facilitate a more nuanced discussion of the 5R’s (relevance, risks, rewards, roadblocks, and repetition) for use with smokers who are unmotivated to quit [54].

### Conclusions

Digital health applications such as TAMI may fill a vital gap in health care by facilitating conversations that evoke a personal argument for change—whether relevant to smoking or other health-related behaviors. The overall structure of focused conversations has been guided by decades of therapy process research, and the clinicians’ utterances that facilitate these conversations are also well established, for example, reflections, affirmations, and open-ended questions [24,29]. While TAMI successfully delivered MI-consistent content using rule-based logic and NLP, future iterations could explore integration with more advanced AI models to improve personalization and conversational fluidity. Although chatbots may never fully replace human interactions and guidance, ever-evolving chatbots may be able to initiate the conversation about change on a broad scale and efficiently facilitate movement through a stepped continuum of care ending with professional clinicians who resolve complex ambivalence toward change.

## Supplementary material

10.2196/88125 Multimedia Appendix 1 Question, reflection, and affirmation library of responses.

## Abbreviations

AI: artificial intelligence
BERT: Bidirectional Encoder Representations From Transformers
CT: change talk
DSDM: dynamic systems development method
MI: motivational interviewing
MICO: motivational interviewing–consistent skills
NLP: natural language processing
RQ: research question
ST: sustain talk
TAMI: technology-assisted motivational interviewing
