# Supplementary material for: Use of the Dynamic Systems Development Method to Inform Technology-Assisted Motivational Interviewing (TAMI) for Tobacco Cessation: Qualitative Study
Source: JMIR Form Res. 2026 Jun 2;10:e88125. doi: 10.2196/88125 (PMC13229398; doi:10.2196/88125)
Supplement: Multimedia Appendix 1 [file formative-v10-e88125-s001.docx]

**Reflections = Strong Sustain Talk**

['Smoking fits with your image of yourself']

['You dont smoke in the car.']

['You have been smoking more because covid19 has made you stressed’]

['You dont mind the price of cigarettes so much.']

['You do have some concerns about your health, but your reasons for smoking carry more weight.’]

['You really enjoy smoking' , 'You arent concerned about heart disease.']

['Smoking calms you down']

['Prescription medications to quit smoking, like Chantix, don't appeal to you', 'You wouldn't want to quit with prescription medications']

['Smoking is a big part of your life. It also sounds like it's such a part of who you are that you will keep smoking no matter the cost.', 'You don't think you can quit', 'You enjoy smoking', 'It sounds like you have given this some thought.']

['Smoking helps you be more social’], [‘You meet more people because of smoking’]

['You seem to have a lot of things going on and smoking seems like a way that you relax and destress','Your smoking seems to be closely related to your stress in life']

['It sounds like you are looking to cope with some of the stress of work', 'You’re looking for moments of relief from work']

['You have other priorities right now']

['Your drinking and your smoking go together','If I'm hearing right, it sounds like you may smoke more when drinking']

['You smoke to keep calm.','Smoking relieves you']

['Smoking helps you cope with anxiety.']

['This is something you don't mind about smoking.']

['You are not concerned with how often you smoke when drinking beer.']

['You are not worried about your blood pressure']

['You smoke when you're bored']

['You do not want a prescription medicine to help with quitting.']

['It feels tough to tell how much smoking increases your risk of cancer. You label your cancer risk as low.']

['You use cannabis, but that isnt related to your smoking.']

['So for you, cardiovascular health is not a big issue.']

['You do not want a prescription medicine to help with quitting.']

['You love the taste of cigarettes’]

['You like your coffee with a cigarette', 'You like to have your cigarette when drinking coffee', 'You don't like to drink coffee without smoking']

['Smoking makes you feel more confident’]

['The coronavirus hasn't changed how you feel about smoking.’]

['You feel dependent on nicotine', 'Nicotine dependence makes it very hard to quit smoking', 'You dont like this feeling of dependence on nicotine.']

['Youre fine with how much you smoke now and arent looking to change’]

[‘You dont feel concerned about how smoking affects your health.’]

['You feel like the best way for you to feel better is by smoking','You rely on smoking to help you feel better’]

['Smoking makes you feel dizzy’]

['Driving just makes you want to have a smoke’]

['This isn't a concern of yours']

[‘You struggle with emphysema.’]

['Smoking gives you a little kick of energy’]

['If I am hearing right, it sounds like your family actually makes it more difficult to quit.']

['You feel more focused when you smoke’]

['The nicotine gum doesn't work for you.']

['You get headaches when you can’t smoke’]

[‘You had to stop while in the hospital’]

[‘Hypertension is not a concern for you’]

['A need for an inhaler does not worry you.']

['You don't care to change your ways with liquor or smoking.’]

['A lozenge will not stop you from smoking.']

[‘Your lungs feel fine even though you smoke’]

[‘The effects of marijuana do not concern you’]

['Nasal spray does not make you want to quit smoking.']

['You enjoy the way nicotine makes you feel’]

['Prescription medications to quit smoking, like Chantix, don't appeal to you', 'You wouldn't want to quit with prescription medications']

['Prescription medications to quit smoking, like Chantix, don't appeal to you', 'You wouldn't want to quit with prescription medications']

[‘You don’t want to try quitting with the patch’]

['Prescription medications to quit smoking, like Chantix, don't appeal to you', 'You wouldn't want to quit with prescription medications']

[‘You have no interest in running’]

[‘Sexual dysfunction is not a concern for you’]

['Your skincare is not a priority for you’]

['You're not bothered by the smell']

['You really like smoking’]

['Smoking relieves your stress’]

['You are not scared of strokes’]

['This doesn't bother you very much’]

['Quitting can be very hard.']

['You think vaping is lame and not a good way to quit’]

['You don’t believe prescription drugs, like varenicline, will help you quit’]

['You enjoy the effects of weed’]

['Quitting smoking could make managing weight more difficult and you care about your weight', 'This is one of your reasons to smoke']

['Weight gain has not been a problem for you’]

[‘Weight loss is not a concern for you’]

['You can’t drink wine without having a smoke’]

['You feel like everybody will get wrinkles eventually’]

['It doesn't sound like the prescription medication Zyban is what you're looking for.']

['You feel like smoking helps you bond with your brother’]

['Smoking doesn't seem to affect your exercise very much.']

[‘You do not smoke in front of children’]

[‘You feel financially stable so the cost of smoking doesn’t concern you’]

['You're not concerned about your smoking right now']

['You still enjoy your cycling']

['Your endurance has not changed much with smoking’]

['You do not correlate smoking with your exercise abilities’]

['You already know about cigarette ingredients and are not worried’]

['You do not have problems with money’]

['You parent’s smoked and so you like to smoke as well’]

['You do not smoke around others so secondhand smoke does not bother you’]

['Smoking with your sister helps you bond’]

['You feel the stigma of smoking is ridiculous’]

['Your strength has not been effected by smoking’]

['You can still enjoy swimming']

['These taxes make things more difficult']

['Smoking doesn't affect your strength']

['Yoga or mindfulness help you manage stress']

['You don't feel like you are addicted to nicotine. So, it seems like there's no reason for you to be concerned about your smoking.', 'Smoking is part of your routine. It's such a pain to change.', 'Smoking cigarettes is an important part of your life.', 'It is difficult to handle the addictive urge to smoke.', 'You're not dependent on nicotine at all.']

**Reflections – moderate Sustain Talk**

['You like how you look when you smoke']

['You are not worried about smoking while driving.']

['COVID 19 makes quitting more difficult.']

['You are fine with how much you spend on cigarettes.']

['Health isn't your only concern right now.']

['Smoking-related heart disease isnt a motivator for you to quit']

['You're looking to cope with some of the challenges in your life', 'You're pushing through these challenges, but they weigh on you', 'Smoking can calm your nerves']

['There is no desire for medication over smoking.']

['This sounds like an ongoing battle almost']

['Your social circle makes it tougher to quit.']

['Stress makes it difficult to quit.']

['Smoking helps you to get through the work day']

['You feel like you have a lot going on and have other things to worry about']

['Drinking affects your decisions to smoke', 'This isn't something you're willing to give up']

['And smoking is one of the ways that you manage those feelings']

['There are these sources of anxiety in your life and smoking helps you escape from them']

['Bad breath is not a problem for you.']

['You enjoy a smoke with your beer']

['Blood pressure can feel like a silent or distant problem, but it still weighs on your mind']

['You smoke to pass the time']

['You would only turn to a quit smoking prescription as a last-resort.']

['Cancer isn't one of your recurring worries. Maybe every once in a while, but you have other things on your mind.']

['Your cannabis use doesnt feel like a problem.']

['There are these downsides to smoking, but they don't quite tip the scales to make you want to quit']

['You would only turn to a quit smoking prescription as a last-resort.']

['You enjoy the taste of cigarettes']

['Cigarettes and coffee go well together for you.']

['Smoking helps with your confidence.']

['COVID hasnt been a factor in your thoughts about quitting.']

['Its hard to resist these strong cravings for nicotine.']

['You dont want to cut down']

['You care about your health, but dont feel like it makes sense to quit smoking at this time.']

['If youre feeling down, cigarettes help you to cope.']

['Smoking helps you feel grounded and stable.']

['You usually smoke when you drive']

['This is something about smoking that you really dont like.']

['Smoking even adds to your concerns about emphysema.']

['If youre feeling down or tired, you turn to smoking for an energy boost']

['Your family can make you need a smoke sometimes.']

['You smoke most when you need to focus.']

['You have doubts about using Nicorette gum.']

['Smoking helps with your headaches.']

['Sorry to hear that you were in the hospital. That must have been tough.']

['Hypertension does not influence your thoughts of smoking.']

['An inhaler does not affect your habits with smoking.']

['Liquor is not a factor in your smoking desire.']

['A lozenge is not going to influence your smoking habits.']

['Your lungs are not a reason to quit smoking.']

['Marijuana is not a reason to stop smoking.']

['Nasal spray does not change your thoughts about smoking.']

['Nicotine is not a reason to quit smoking.']

['NRT is not helpful for quitting.']

['The patch is not changing your opinion of smoking.']

['There is no desire for a prescription over smoking.']

['Running does not stop your smoking.']

['Sexual dysfunction does not stop your smoking habits.']

['Skin care does not influence your smoking.']

['Smell does not stop you from smoking.']

['Smoking is not a problem in your life.']

['For you, smoking helps with this.']

['Strokes are scary.']

['You like to take care of your teeth']

['Triggers make it that much more difficult to quit.']

['You enjoy vaping.']

['You might be interested in other methods to quit instead, like the patch or quit-smoking counseling.']

['You enjoy cannabis. It sounds like you may also like mixing cannabis with tobacco.']

['You would like to manage your weight']

['For you, wine and cigarettes go together.']

['The idea of wrinkles don't bother you so much.']

['You might be interested in other methods to quit instead, like the patch or quit-smoking counseling.']

['So, your family might be able to make an impact here.']

['Smoking seems to affect your exercise and fitness']

['It sounds like you value the health of the children around you']

['Smoking is expensive.']

['COVID-19 can be scary and smoking doesn't make it any less scary.']

['Cycling gives you a feeling of freedom, but smoking might get in the way.']

['It doesn't sound like smoking affects your physical endurance very much']

['It doesn't sound like smoking affects your ability to exercise very much.']

['You're unbothered by the ingredients of a cigarette.']

['Smoking is definitely expensive.']

['This isn't a reason you would want to quit.']

['You try to be mindful of where you smoke.']

['So, your family might be able to make an impact here.']

['The stigma surrounding smoking can be hurtful.']

['Your strength has not been effected by smoking’]

['You can still enjoy swimming']

['These taxes make things more difficult']

['Smoking doesn't affect your strength']

['Yoga or mindfulness help you manage stress']

['While not ideal, you don't mind being dependent on nicotine. It also seems like you may not feel addicted to cigarettes at all.', 'You might feel that whether or not cigarettes are addictive doesn't matter.', 'You could quit whenever you want to and there's nothing wrong with how things are now.', 'You don't feel as if you have a problem.', 'You have other priorities right now.']

**Reflections: Neutral**

['You've thought about how smoking affects your image and appearance']

['Smoking and driving isn't something you've thought much about.']

['COVID-19 hasn't changed your willingness to quit. ']

['You certainly dont enjoy how expensive a habit smoking is']

['You can feel how smoking affects your body']

['You arent all that concerned about your heart health.']

['Your mental health affects your smoking habits.']

['You've considered a prescription medicine to help you quit smoking']

['You have been thinking about smoking cessation.']

['With the support of people in your life, quitting can be easier']

['Stress can be a barrier to quitting for you.']

['For you, work might be a trigger to smoke.']

['It is difficult to think about how smoking makes us age faster']

['Your smoking habits don't change at all when you drink']

['Anger is such an unpleasant feeling and it sounds like smoking helps this feeling go away']

['There are these anxieties, but you are open to addressing them in other ways instead of smoking']

['You care about your dental hygeine and breath.']

['Drinking beer is not at all related to smoking for you']

['You don't have problems with your blood pressure']

['You don't smoke more when bored']

['When it comes to using a quit-smoking medication or not, you could go either way.']

['Smoking-related cancer is something that you worry about.']

['Your tobacco smoking is completely separate from your cannabis smoking']

['If you do decide it quit, it would be for other reasons.']

['When it comes to using a quit-smoking medication or not, you could go either way.']

['The taste of cigarettes does not matter much to you']

['You enjoy drinking coffee']

['You're feeling confident about this.']

['COVID might have made you think more about quitting, but it hasn't tipped the scales one way or the other.']

['When thinking about potentially quitting, these strong cravings to smoke are standing in the way and you are looking for a way to manage them.']

['You are on the fence about cutting down. There are these things you like about smoking and you also have some concerns.']

['You care about your health and longevity.']

['When you are struggling, a cigarette provides you with comfort']

['You dislike the side effects of smoking']

['Youre very self-aware about this.']

['You would rather not have this problem']

['Your emphysema concerns motivate you you to take care of your lungs.']

['You like that boost of energy']

['You have the support of your family']

['On one hand, there are these concerns about staying focused, and then on the other you have also expressed your concerns about smoking.']

['You don't really have an opinion about chewing gum instead of smoking. ']

['These headaches can be a real pain.']

['That must have been stressful being in the hospital and also not being able to smoke']

['Hypertension has a factor in considering your habits with smoking.']

['Inhalers can influence your habits with smoking.']

['Liquor may affect your smoking.']

['Lozenges may change your use of cigarettes.']

['Your concern for your lungs affect your thoughts about cigarettes']

['Your relationship with marijuana has a roll in your relationship with cigarettes.']

['Nasal spray affects your breath and smoking.']

['Nicotine influences your smoking habit.']

['You've considered a prescription medicine to help you quit smoking']

['The patch can influence a decision to quit.']

['You've considered a prescription medicine to help you quit smoking']

['Running influences your smoking habit.']

['Placeholder complex reflection']

['Skin care affects your smoking habits.']

['As a smoker, you hear people talk about the smell of cigarettes']

['You've been thinking about the effects of smoking.']

['Stress can be a barrier to quitting for you.']

['Avoiding strokes may be a reason to quit for you, but it isn't a huge factor.']

['Dental hygiene may be a reason to quit for you, but it isn't a huge factor.']

['You've thought about triggers to smoke and potentially some ways to avoid them.']

['Vaping doesn't seem to affect your smoking one way or the other.']

['You're open to exploring prescription drugs to assist with quitting']

['You don’t use tobacco and cannabis at the same time.']

['You use cigarettes to manage your weight']

['You may be worried about weight gain after quitting']

['You tend to smoke more when you drink.']

['You've given some thought to how smoking affects your skin.']

['You're open to exploring prescription drugs to assist with quitting']

['Your family might be a helpful resource if you asked for support.']

['You've thought about this, but it doesn't seem to push you one way or the other.']

['You may have considered how smoking affects the children around you.']

['The cost of cigarettes bothers you, but isn't one of your main reasons to quit.']

['The COVID-19 pandemic seems to have raised new thoughts about smoking.']

['You don't notice any negative effects on your cycling']

['You don't feel any negative impacts from smoking on your exercise.']

['Occasionally, you might think about the ingredients in a cigarette.']

['The cost of cigarettes may be high, but this isn't one of your reasons to quit.']

['Your family might be a helpful resource if you asked for support.']

['You've thought about the effects that smoking has on others.']

['Your family might be a helpful resource if you asked for support.']

['Smoking is frowned upon by society and that feeling can be isolating at times.']

['Paying more in taxes is never fun.']

**Reflections – Moderate Sustain Talk**

['You care about your image']

['You'd prefer not to smoke while driving']

['COVID19 makes you want to quit smoking.']

['Cigarettes are very expensive.']

['You seem to be aware of how smoking can affect your health', 'You have some reasons to stop smoking', 'You notice how smoking affects your health', 'Your priorities are shifting towards your health']

['You want to limit your risks of getting heart disease.']

['Your mental health makes you consider quitting smoking.']

['You're willing to consider medication to consider quitting smoking.']

['You are considering smoking cessation and the value it adds to your life.']

['The people in your life can motivate you as well.']

['Work can be a trigger to smoke for you']

['There are some ways to manage work-related cravings, like chewing nicotine gum, going for a quick walk, or playing a phone game. You've got some good ideas for this too.']

['You want to be able to feel young and healthy for as long as you can']

['You're mindful of how alcohol affects your smoking.']

['Managing these feelings can be difficult, but you sound open to managing them without turning to a cigarette.']

['Anxiety can be so difficult. Its no wonder why many people say that they smoke to manage their anxiety. For you, it sounds like this may be true, but that you also know other ways to manage anxiety.']

['You enjoy having fresh breath.']

['You'll enjoy a beer, but it sounds like you want to be mindful of your smoking when you are drinking.']

['You are worried about how smoking affects your blood pressure.']

['Boredom can definitely be a trigger for many people. It may be a trigger for you, but it seems like you might have a few ideas on how to manage boredom without smoking.']

['A quit-smoking prescription medicine might be a good fit for you.']

['Im hearing that you dont want cancer and that it might be one of your biggest reasons to quit',]

['It sounds like your cannabis smoking and cigarette smoking might be related']

['This sounds like one of your reasons to quit']

['A quit-smoking prescription medicine might be a good fit for you.']

['The taste of cigarettes is not one of the things you like most about smoking']

['Coffee can be a trigger to smoke for many people']

['You feel confident that you could quit if you wanted to.']

['The coronavirus has made you more interested in quitting.']

['Nicotine cravings seem to stress you out quite a bit.', 'Cravings feel like an unpleasant itch that you just have to scratch.']

['You would like to cut down on your smoking.']

['You want to quit to save your life.']

['You dont like how much you smoke']

['You care about your health.']

['You may also have a few ideas on how to address this.']

['You want to feel more confident and quitting may help with that']

['The health of your lungs is important to you.']

['Smoking makes you feel a short term boost to energy, but leads to a crash. You want out of this cycle.']

['You want to be able to be there for them']

['You want to be able to focus without turning to a cig']

['You'd be willing to try gum instead of smoking.']

['Smoking may be giving you headaches.']

['No one likes being in the hospital, but its even tougher if you have bad cravings to smoke but cant.']

['Improving hypertension may be an incentive for you to quit.']

['Inhaler usage can play a roll in wanting to quit smoking.']

['Liquor affects your desire to quit smoking.']

['Lozenges affect your desire to quit.']

['Your lungs influence your consideration for smoking.']

['Marijuana makes you consider changes in smoking.']

['Nasal spray supports your desire to quit.']

['You desire some change with nicotine and your smoking habits.']

['You hope NRT and other methods could support a change.']

['A patch can support your desire to change smoking habits.']

['You are willing to consider prescriptions to consider quit smoking.']

['You're willing to consider changes in smoking for running.']

['You're willing to explore changing smoking habits because of sexual dysfunction.']

['You desire to change your skin care and lung care with changes in smoking.']

['You value how you appear to others']

['You are considering changes in smoking.']

['Work can be a trigger to smoke for you']

['The idea of a stroke may be a reason to quit for you.']

['You want to take care of your teeth.']

['You're able to identify triggers to smoke before they happen.']

['It sounds like you're considering vaping nicotine instead of smoking cigarettes to help with cravings.']

['You've considered quit-smoking prescriptions, like Varenicline']

['You're thinking creatively about ways to quit.']

['Weight management is important to you, but you're willing to look at other ways to manage your weight without smoking']

['You can prepare for cravings by avoiding alcohol, or keeping smokes away from you while you drink.']

['You don't want smoking-related skin issues, like wrinkles.']

['You've considered quit-smoking prescriptions, like Zyban.']

['Your family can motivate and support you.']

['You're noticing an example of how quitting can improve your physical health']

['The children in your life help motivate you to make this change. ']

['You don't like how expensive cigarettes have gotten.']

['COVID-19 may have changed your perspective on smoking.']

['You're noticing an example of how quitting can improve your physical health']

['You're concerned about all of the dangerous ingredients in cigarettes and tobacco.']

['Smoking can definitely be expensive. Quitting would save you a lot of money. ']

['Either being a parent, or being cared for by a parent is a powerful motivator to quit.']

['You don't want to expose other people to secondhand smoke.']

['Your family can motivate and support you.']

['The stigma surround smoking can be harsh, but you have your own reasons to quit.']

['Saving money is a strong reason to quit.']

['You're noticing an example of how quitting can improve your physical health']

**Reflections: Strong Change Talk**

['You don't like how smoking affects your image', 'This matters to you.']

['Quitting will also help keep the car clean']

['COVID19 makes quitting more important than ever. ']

['You are concerned about the financial costs of smoking.', 'Saving money is important to you.', 'Cigarettes are a financial burden.']

['You know it's not healthy', 'You look forward to improved health and an improved life']

['You have some concerns about smoking-related diseases', 'This is really important to you', 'Quitting is the best thing you can do to have a healthy heart.']

['Although your life has many stressors, you're on your way to making sure that nicotine dependence is not one of them']

['You're willing to try medication and you're ready to make changes with your smoking.']

['These threats to your health make you want to quit', 'You want to quit altogether ', 'You don't see yourself quitting', 'You're looking for steps toward a healthier life']

['You have a group of people that can support you', 'You care about the example you set for others']

['You're looking for a healthier option']

['You are able to handle your work related-stress in other ways.']

['The thought of growing older feels tough as is. It sounds like smoking adds to this worry.']

['You know best how to manage your smoking when you drink.', 'Not smoking when you drink can be tough, but you feel up to the task.']

['Quitting sounds important to you.']

['The act of smoking feels like it relieves stress in the short term, but it sounds like it adds even more stress and concern in the long run']

['This seems like another one of your reasons to quit']

['You enjoy drinking a beer']

['You have concerns about your blood pressure, and it sounds like smoking is making things worse.']

['Even if smoking helps with passing the time, for you, its just not worth the cost']

['From what I gather, it sounds like you are open to many strategies to quit--including a quit-smoking prescription like Bupropion.']

['Cancer is scary and you want to greatly reduce your cancer-risk.']

['On one hand, you enjoy using cannabis, but it also sounds like you want to cut down on your tobacco smoking.']

['You want to a healthy heart and body.', 'This is quite important to you']

['From what I gather, it sounds like you are open to many strategies to quit--including a quit-smoking prescription like Chantix.']

['You hate the taste of cigarettes']

['Coffee can certainly be a trigger to smoke']

['You are confident about this.']

['The coronavirus makes quitting smoking feel more important now than ever.']

['I think its great that you are motivated to live a long and healthy life. It sounds like you are thinking of giving quitting another try.']

['When times get tough, you want to find support rather than bring yourself down with smoking.']

['Driving--especially in traffic-- can be a big trigger for you.']

['This is concerning to you.']

['The damage that smoking has done to your lungs has crossed a line and you want to make a change.']

['Smoking feels like it zaps your energy']

['You don't want your smoking to affect your family.', 'Family and loved ones can be a powerful motivator to quit.']

['You may be open to learning ways to help you focus that dont involve smoking.']

['You are hopeful that the nicotine gum could help you quit smoking.']

['These headaches sound really troublesome.']

['You dont want to wind up in the hospital.']

['Your health and healing hypertension by managing your smoking habits are a priority.']

['Issues with inhalers and quitting smoking habits are important for you.']

['Managing liquor and cigarettes is something you care about changing.']

['Consuming lozenges and cigarettes are a concern for you to change.']

['You care about your lungs and want to change your smoking habits.']

['You care to change your relationship with marijuana and cigarettes.']

['Making shifts with nasal spray support your desire to make big shifts with quitting smoking.']

['You desire to overcome nicotine and smoking habits.']

['You're ready for NRT and other methods to kick the smoking habit.']

['You want to use a patch or any other method because you are ready to stop smoking.']

['You're willing to try other medicine, because you are stronger than tobacco.']

['You're ready to run without smoking in your life.']

['You want to make changes for sexual dysfunction and you're ready to quit smoking.']

['You care about the health of your skin and lungs and want to quit smoking.']

['Your appearance is important to you', 'You like to take care of yourself']

['Making shifts with smoking is important to you.']

['You're looking for a healthier option']

['You want to minimize your risk of having a stroke.']

['Caring for your teeth is a great reason to quit.']

['You're ready and able to handle cravings if you decide to quit.']

['Vaping is something you use to cut down or quit.', 'For you, vaping helps with the cravings']

['Quit-smoking medications can be very helpful.']

['You seem to be giving quitting tobacco some serious thought.']

['You want to make a change and you're able to manage your concerns about weight in other ways', 'You're ready to do this.']

['It sounds like quitting smoking is very important to you.']

['You're identifying some of the challenges to quitting, as well as some ways to overcome them.']

['You want healthy skin.']

Quit-smoking medications can be very helpful.

['You have support throughout this process']

['You see how quitting would improve your general health.']

['You want to be a good example.']

['Quitting smoking saves a lot of money.']

['COVID-19 has been a strong motivator for you.']

['You see how quitting would improve your general health.']

['You worry about the ingredients in a cigarette.']

['Smoking is just too expensive.']

['If you decided to quit, the people in your life would also benefit too.']

['You have support throughout this process']

['Smoking takes an emotional toll, too.']

['You see how quitting would improve your general health.']

['Quitting would also relieve some financial stress.']

['You see how quitting would improve your general health.']

['Yoga and Mindfulness are powerful tools to help folks quit. It sounds like you may be considering them.']

**Questions – Strong Sustain Talk**

['Quitting smoking puts a lot of money back in your pocket. What would this mean for you?']

['How will your health get better when you quit smoking?']

['How would your life get better when you quit?']

['What are the next steps for you?']

['How could your family support you in quitting?']

['How could the people in your life support you through quitting?']

['Would quitting smoking change the way you view yourself?']

['What would subtracting smoking add to your life?']

['How does smoking effect your mental health?']

['Tell me more about this. How do you think it relates to your smoking?']

['If you took quit-smoking medications to manage your cravings and withdrawal symptoms, what else would you need to support you? A successful quit often involves using many strategies.’]

['How does this affect your desire to quit?']

['In what ways could nicotine replacement products (like the patch, or nicotine gum) help you?']

['If you took quit-smoking medications to manage your cravings and withdrawal symptoms, what else would you need to support you? A successful quit often involves using many strategies.’]

['If you took quit-smoking medications to manage your cravings and withdrawal symptoms, what else would you need to support you? A successful quit often involves using many strategies.’]

['What could you do with more time? Quitting smoking gives you more time each day, but it also can help you live a longer, healthier life. What would this look like for you?']

['Does alcohol make it hard to quit smoking? If so, why? If not, what other things make it difficult to quit?']

['How would quitting affect how you feel when you are not smoking? Let's assume that the temporary withdrawal symptoms have faded and are a thing of the past.']

['In what ways could quitting aid with your anxiety?']

['How does smoking effect your hygiene?’]

['What problems arise when you pair beer with smoking?']

['It sounds like your blood pressure is a concern for you. How could quitting help with this?']

['What do you like to do outside of smoking?’]

['How would your brother feel if you were to quit smoking?’]

['What are your thoughts about the effects of cannabis?']

['In what ways could your cardio improve if you quit?']

['How important is improving your cardiovascular endurance to you?']

[‘Are there precautions you take with smoking when children are around?’]

['How will your life improve without cigarettes?']

[‘What are some ways you start your morning?’]

['How will your confidence change when you quit smoking?']

['How has the coronavirus affected your smoking?’]

['It looks like the cost of cigarettes bother you. How will this benefit you when you quit?']

['Covid-19 is giving you some concerns about being a smoker. How would quitting help you during this time?']

['How can you help lower cravings?’]

['How has cutting down worked for you or anyone you know in the past?']

['How would quitting help you with cycling?']

[‘Could you think of anyone that would be heavily affected by your death?’]

['How important is your mental health to you?']

['Could you elaborate on your dizziness? In what ways would quitting effect this?']

[‘Do you have any concerns about smoking in the car?’]

['How important is reducing empysema to you?']

[‘How has smoking affected your endurance?’]

['What do you think quitting smoking would do to your energy levels throughout the day?']

[‘Can you see the effects of smoking when you are exercising?’]

[‘What are some things that help you stay focused?’]

['What are your thoughts on nicotine gum for quitting?']

['Could you expand on information about your headaches? How would this change if you quit smoking?']

['In what ways are you concerned about hospitalization?']

['How concerned are you about hypertension when it comes to smoking?']

['What ingredients in cigarettes raise the most concern for you?']

['What does the idea of carrying an inhaler mean to you?']

['How does the relationship between liquor and smoking effect you?']

['What are your thoughts on lozenges as a method of smoking cessation?']

['Can you think of activities you would be able to do if your lungs improved?']

['What are your thoughts on the effect of marijuana?']

['What could you buy with the money you spent on cigarettes if you quit?']

['How do you feel about nicotine nasal spray as a way to quit?']

['What are your concerns about nicotine in cigarettes?']

['How do you think nicotine replacement therapy would help you in quitting?']

['In what ways could your parents support you if you quit smoking?']

['How has the patch worked for your or someone you know if the past?']

['What are your thoughts on prescription medication for smoking cessation?']

['How would your running improve if you quit smoking?']

['How does this fit into the feelings you have about quitting?']

['How would this issue improve if you quit smoking?']

['How would your sister feel about you quitting?']

['How would your skin improve if you quit smoking?']

['What are the worst things about smoking?']

['How does the stigma of smoking bother you?']

['How would your strength be effected if you quit smoking?']

['How concerned are you about strokes?']

['How would quitting help with swimming?']

['It seems like the taxes that come with smoking bother you. Can you think of ways quitting would help you financially?']

['What would the next steps be for you if you wanted to quit?']

['What actions can you take to prevent the triggers to smoke?']

['For some people, vaping leads to more cigarette smoking. For other people, vaping can help reduce cigarette smoking. What are your thoughts on vaping?']

['What concerns do you have about smoking weed?']

['What are ways you could prevent weight gain if you quit smoking?']

['What are some actions you can take to address this concern without smoking?']

['What aspects could help with weightlifting if you quit smoking?']

['What are other things you can enjoy instead while drinking wine?']

['How would quitting change your appearance?']

['How might yoga or other mindfulness techniques help you?']

**Questions – Moderate Sustain Talk**

['Without the need for cigarettes, what would you buy instead?']

['How has smoking changed the way you think about your health?']

['It sounds like you may be thinking about quitting. What do you think is keeping you from trying?']

[You see the link between smoking and your weight. What are some things you could change?']

['What would quitting smoking do for your heart health?']

['How would your family react if you quit?']

['How could your social circle help you with quitting?']

['In what way can you prevent the smell from affecting your life?']

['How would quitting affect your day-to-day life?']

['How would quitting benefit your overall health?']

['In what ways could quitting help improve your mental health in the long run?']

['What could this tell you about your smoking?']

['Quit-smoking prescriptions, such as Bupropion, have been shown to greatly reduce cravings. Would this be an option that you would consider? Why or why not?']

['Quit-smoking prescriptions, such as Chantix, have been shown to greatly reduce cravings. Would this be an option that you would consider? Why or why not?']

['How might quitting make this issue better for you?']

['How could the patch or nicotine gum help you quit?']

['Quit-smoking prescriptions, such as Varenicline, have been shown to greatly reduce cravings. Would this be an option that you would consider? Why or why not?']

['Quit-smoking prescriptions, such as Zyban, have been shown to greatly reduce cravings. Would this be an option that you would consider? Why or why not?']

['What would you tell a young smoker?']

['What are some ways you can manage your tobacco cravings while drinking?']

['For some people, smoking can give them a temporary sense of calm when they are angry, but make them feel frustrated whenever they have a craving. On the whole, do you think smoking makes you feel more or less angry? Do you feel in control?']

['How could quitting help you with anxiety?']

['How could quitting help your breath?']

['How could quitting help your beer drinking?']

['How could quitting help your blood pressure?']

['How could quitting help your boredom?']

['How could your brother support you in quitting?']

['How would quitting tobaccco fit into your cannabis smoking?']

['How could quitting help your cardio?']

['What are ways you can quit smoking to help your heart health?']

['How could your children support you in quitting?']

['What are some methods that you can use to quit smoking cigarettes?']

['How could quitting make drinking coffee more pleasurable in the long run?']

['How would quitting affect your confidence?']

['How might quitting help stop the spread of coronavirus?']

['What are the ways you can quit smoking and reduce the cost of cigarettes?']

['How might quitting help stop the spread of coronavirus?']

['What ways would you consider managing craving and quitting?']

['How would you consider cutting down?']

['How does cycling make you consider quitting?']

['How does the idea of death make you consider quitting?']

['In what way does depression influence a desire to change smoking habits?']

['How does your experience with dizziness make you consider changes?']

['In what ways does diriving influence your desire to quit?']

['How do your thoughts on emphysema make you consider change?']

['In what way does endurance play a role in thoughts of quitting?']

['For many people, smoking gives a short-lived burst of energy. If you wanted to, how else could you get that burst of energy without smoking?']

['In what way does exercise affect your desire to quit smoking?']

['How can focus be directed in changing your relationship with smoking?']

['In what ways can gum support your change in habits?']

['How do headaches affect your consideration for change?']

['How do your thoughts on hospitilization impact your desire for change?']

['In what way does hypertension influence your consideration for quitting smoking?']

['In what ways do ingredients affect your consideration for change?']

['How does an inhaler influence your decisions for smoking cessation?']

['How do you want to consider liquor in relationship to changes in smoking?']

['How can lozenges affect a decision for quitting?']

['In what ways do lungs influence a consideration for ending smoking habits?']

['Could smoking cannabis/marijuana make quitting more difficult for you?']

['If you had all the money back that you spent on smoking, what could you buy for yourself?']

['Would you be open to trying the nicotine nasal spray?']

['How does nicotine influence your life?']

['What have you heard about nicotine replacement therapy (also known as the patch, gum, and lozenge)?']

['How did your parent initially react when you told them about your smoking?']

['What could get in your way of using the patch if you wanted to? How could you manage that situation?']

['Why might quit-smoking medications be helpful for some folks?']

['How could quitting help your cardio?']

['Do the people you live with make it more difficult to quit?']

['How do you feel about this?']

['How could your sister support you in quitting?']

['How is your skin affected by smoking?']

['What would have to change to turn the idea of quitting into a reality?']

['This stigma around smoking can be tough to manage. How could the people in your life be more supportive of you?']

['Months afer someone quits, their breathing and cardiovascular health greatly improve. What would this mean for your day to day life?']

['Quitting is one of the best things you can do to decrease your risk of a stroke. How do you feel about this?']

['How could quitting help your cardio?']

['How much would you save each month if you quit?']

['How does smoking harm your teeth?']

['What's your worst trigger to smoke and what could you do to avoid it? A trigger is something that makes you have a strong craving to smoke.']

['For some people, vaping leads to more cigarette smoking. For other people, vaping can help reduce cigarette smoking. What are your thoughts on vaping?']

['How would quitting tobaccco fit into your cannabis smoking?']

['What are the best ways to manage concerns about weight?']

['Would you be able to enjoy wine more after you quit?']

['How else could quitting help your skin?']

['How can yoga help you with quitting?']

**Questions – Neutral**

['And how does that relate to your smoking?']

['What are your health concerns about smoking?']

['What do you think are the best reasons to quit smoking?']

['How have you managed your weight in the past?']

['How do you feel about this?']

['What would you quitting mean for your family?']

['What do you miss out on when you smoke?']

['Is the smell of cigarettes something that you think often about?']

['In what ways does smoking make you feel more stressed?']

['Is this a reason to quit for you?']

['What other options you could have to help with this--aside from smoking?']

['How would you relate this to your smoking?']

['How do you feel about using a quit-smoking prescription medication to quit?']

['How might quitting make this issue better for you?']

['How do you feel about the patch?']

['How do you feel about using a quit-smoking prescription medication to quit?']

['What affects can smoking have on the aging process?']

['Do drinking alcohol and smoking go hand-in-hand for you?']

['How might quitting smoking help with your anger?']

['How might quitting smoking help with your anxiety?']

['How might quitting smoking help with your breath?']

['How might quitting smoking help with your beer drinking?']

['How might quitting smoking help with your blood pressure?']

['How might quitting smoking help with your boredem?']

['What has your brother mentioned to you about quitting smoking?']

['How might quitting smoking help with your cannabis use?']

['What would quitting smoking mean for your breathing?']

['What would help you consider quitting to improve heart health?']

['How do children factor into your thoughts about quitting?']

['How would life be different without cravings for nicotine?']

['How could quitting make drinking coffee more pleasurable in the long run?']

['What would quitting mean for your confidence?']

['Has the coronavirus affected your thoughts on quitting smoking?']

['What would help you consider quitting to help you cut costs?']

['Has COVID-19 affected your thoughts on quitting smoking?']

['How could quitting help manage your craving issues?']

['How does cutting down help?']

['How would quitting smoking improve cycling?']

['What are some of the ways quitting would improve your thoughts on death?']

['How would quitting smoking affect depression?']

['How would a change in smoking affect dizziness?']

['In what ways would smoking cessation have an effect on your driving habits?']

['How does smoking habits improve chances with emphysema?']

['What are some of the ways quitting could influence endurance?']

['How does a craving for a cigarette affect your ability to focus at work?']

['What are some ways in which smoking cessation infleunce exercise?']

['How does a change in smoking habits affect focus?']

['How is gum involved in quitting smoking?']

['In what ways does quitting improve issues with headaches?']

['How would quitting be affect hospitilization?']

['In what ways does a change in smoking improve hypertension?']

['In what ways does smoking cessation affect your experience with ingredients?']

['How would quitting improve inhaler usage?']

['In what ways does smoking cession affect engagement with liquor?']

['How does quitting relate to lozenges?']

['What are some ways smoking cessation could improve your lungs?']

['What does this mean for you?']

['How might quitting fit into your larger goals?']

['What would stop you from tying the nicotine nasal spray?']

['What would cutting down on nicotine help with your autonomy and self-control?']

['Why might nicotine replacement products like the patch help some people quit?']

['How could your family support you if you wanted to quit? Do you think they would be very helpful?']

['Where do you think you could get more information about the patch?']

['How do you feel about using a quit-smoking prescription medication to quit?']

['What would quitting smoking mean for your exercise routine?']

['How could you quitting smoking help others?']

['How does this factor into your feelings about smoking?']

['How might quitting be easier with a team of support on your side?']

['What would clearer skin mean for you?']

['Tell me more about a time in the past when you wanted to quit.']

['What would you tell someone who is thinking about quitting, but feeling pessimistic?']

['Quitting can be tough to do. But if you keep at it, you will eventually succeed. What could you draw strength and support from in this fight?']

['How would reducing your risk of a stroke feel for you?']

['What would quitting smoking mean for your exercise routine?']

['Aside from the cost of the pack, what are the hidden costs of smoking? How could smoking make you pay more in the future?']

['What has your dentist mentioned about smoking in the past?']

['How have you managed cravings in the past?']

['How does vaping relate to smoking tobacco?']

['How might quitting smoking help with your cannabis use?']

['How might smoking actually make weight loss more difficult?']

['How does wine relate to smoking in your case?']

['Woud you enjoy improved skin health if you quit?']

['Has yoga been helpful for you in the past?']

**Questions – Moderate Change Talk**

['What are your financial concerns?']

['How does smoking affect your health?']

['You may have gone without cigarettes for a week or more. What were the good things about that time?']

['What is the connection between smoking and your weight?']

['What are some of your health concerns?']

['What effects does your smoking have on your family?']

['How has your smoking affected your social life?']

['In what way has the smell of cigarettes affected your life?']

['When you feel stressed, how does that affect your smoking?']

['Have health issues ever become a reason to quit before?']

['How have these concerns affected your interest in quitting?']

['How would you relate this to your smoking?']

['Have you tried a quit-smoking medication in the past? If you did, how did it go? If not, what would make you consider trying one?']

['Have you tried a quit-smoking medication in the past? If you did, how did it go? If not, what would make you consider trying one?']

['How might quitting make this issue better for you?']

['How do you feel about nicotine gum?']

['Have you tried a quit-smoking medication in the past? If you did, how did it go? If not, what would make you consider trying one?']

['If you could go back in time, would you start smoking? Why or why not?']

['For folks who have quit, alcohol can sometimes be a huge trigger to start smoking again. What realistic advice would you give them?']

['And how does that relate to smoking for you? ']

['If you wanted to, what could yo do while drinking to minimize your risk of smoking?']

['How would quitting smoking help with blood pressure?']

['How could you entertain yourself without cigarettes?']

['If you asked your brother for support when quitting, how do you think it would go?']

['For you, if you were to quit, would you quit both cannabis and tobacco? Or would you just prefer to only quit tobacco?']

['What are some of the pros of quitting?']

['What does this mean for you?']

['How would (or do) your parents feel about your smoking?']

['How do cigarettes help you?']

['What else could you pair your coffee with?']

['How would quitting smoking help with your confidence?']

['How would quitting smoking help with your chances of not being affected by cornovirus?']

['How would quitting smoking help with costs?']

['How would quitting smoking help with your chances of not being affected by cornovirus?']

['What would help with cravings and quitting?']

['What would make you consider cutting down or quitting?']

['What's needed for your cycliing experinece to consider a change in smoking?']

['How would your experience with death affect your consideration for quitting?']

['What would help depression or a consideration for quitting?']

['What would need to change with dizziness to change smoking habits?']

['How could driving changes make you consider change with smoking?']

['What would need to happen with emphysema for you to consider changes?']

['What about endurance would help you consider quitting smoking?']

['Some people observe that they have more energy after quitting smoking. What could you do with this extra energy?']

['How would your experience with exercise need to shift for you to quit smoking?']

['How would changes in focus change your thoughts on your smoking behavior?']

['What about gum or other changes be needed for you to think about quitting?']

['In what ways can headaches or other shifts make you consider making a change with smoking?']

['What would need to happen with hospitalization for you to consider quitting?']

['What needs to happen with hypertension for you to consider quitting smoking?']

['How can ingredients change for you to consider changing your habits with smoking?']

['How can inhalers cause you to consider quitting smoking?']

['What are things that need to happen with liquor in order for you to think about changing lifestyle with cigarettes?']

['In what ways can lozenges help you evaluate what needs to be done in order for you to think about quitting cigarettes?']

['What would you need to happen with your lungs in order to even consider changing habits with smoking?']

['What does this mean for you?']

['What would have to change for you to consider quitting?']

['What are the best alternatives to control cravings for you?']

['How might nicotine has some control over you?']

['If you didn’t have cravings, or they were less severe, how would that affect your thoughts about quitting?']

['What would you say is keeping you from quitting right now?']

['The patch can be purchased at most pharmacies over the counter. There are also some programs that give you patches for free to help you quit. What could the patch do for you?']

['Have you tried a quit-smoking medication in the past? If you did, how did it go? If not, what would make you consider trying one?']

['What would better breathing and stamina mean for your exercise routine?']

['What are the downsides to smoking around others?']

['What does this mean for you?']

['Who do you have on your side to support you?']

['How do you feel about the affect that smoking has on your skin?']

['Tell me more about a time in the past when you wanted to quit.']

['Why is smoking so stigmatized by the general community?']

['Quitting can be tough to do. But if you keep at it, you will eventually succeed. What could you draw strength and support from in this fight?']

['How would a stroke change your life?']

['What would better breathing and stamina mean for your exercise routine?']

['Aside from the cost of the pack, what are the hidden costs of smoking? How could smoking make you pay more in the future?']

['How does smoking affect your palate?']

['How can you avoid triggers better?']

['Are there other ways to manage cravings besides vaping?']

['For you, if you were to quit, would you quit both cannabis and tobacco? Or would you just prefer to only quit tobacco?']

['How might smoking actually make weight loss more difficult?']

['Would you be open to exploring food pairings with wine instead of cigs?']

['What are some other concerns you may have?']

['How could the teachings of yoga be applied to quitting?']

**Questions – Strong Change Talk**

['What other costs of smoking can you think of?']

['What would have to happen for you to consider quitting smoking?']

['What do you think are the best reasons to quit smoking?']

['What strategies could you use to manage your concerns about weight? ', 'What have you done in the past to help you with your weight? ', 'What have others done to manage their weight?']

['What are your thoughts on the other health effects of smoking?']

['How does smoking affect your family and your living situation?']

['What would it take for you to feel supported in quitting?']

['What would you say is the biggest downside to smoking?']

['Stress can definitely make it feel difficult to quit smoking. What other ways--besides smoking--do you think you can manage your stress?', 'How have you managed stress in the past?']

['How have you managed your health in the past?']

['How have you managed mental health concerns in the past?']

['How would this relate to your smoking?']

['Have you tried a quit-smoking medication in the past? If you did, how did it go? If not, what would make you consider trying one?']

['How might quitting make this issue better for you?']

['Have the patch or nicotine gum helped you in the past?']

['Have you tried a quit-smoking medication in the past? If you did, how did it go? If not, what would make you consider trying one?']

['What do you like about smoking?']

['Could you think of any pleasurable substitute for smoking? Whether you're ready to quit or not, its always good to think of healthy things that make you happy.']

['How do you usually cope with anger?']

['What are some techniques that have helped with your anxiety in the past?']

['What do you know about smoking and dental health?']

['How does your drinking affect your smoking?']

['What would have to be different to change your mind about this?']

['What are some other activities you could do when you are bored?']

['How could your brother help you quit?']

['Would you say that your cannabis smoking is related to your tobacco smoking?']

['How else might your life improve if you quit?']

['What are your thoughts on laws that prevent secondhand smoke in crowded places?']

['What advice would you give to someone who is thinking about smoking for the first time?']

['Might there be another reward you can associate with your coffee, aside from your smoke?']

['How does smoking relate to your confidence?']

['How has the coronavirus affected your feelings about quitting--if at all?']

['How much money would you guess that you spend on cigs in a year?']

['What other techniques could you use to quit?']

['Althought you don't want to quit and manage cravings right now, what would need to happen for this to change?']

['Even though you don't want to cut back now, what would need to happen for you to consider quitting smoking?'"

['Even though you don't need to quit smoking now to help you with cycling, what would need to change for you to consider changing smoking habits?']

['Even though death is not upon you and you don't need to quit right now, what would need to happen for you to consider quitting?']

['Although you don't want to quit to manage depression now, what would you need for you to consider a change in smoking?']

['You don't feel quitting or dizziness is an issue to address now, however is there something you need in order to consider changing?']

['Although you don't need to quit to improve driving, what would make you even consider a change in smoking?']

['You're not ready to quit smoking to improve issues with emphysema now. Are there other things that you need addressed to ever consider quitting?']

['Although you don't need to quit and improve endurance now, what would need to change for you to consider quitting smoking?']

['Although a smoke might feel invigorating, quitting smoking has been shown to improve overall energy levels within three weeks. What are some other ways to keep your energy levels up throughout the day?']

['Although you don't need to quit in order to exercise, what changes would make you consider quitting?']

['Regardless of this not being a time to change your focus or behaviors with smoking, what changes would need to happen in order for you to consider a change in smoking?']

['Even though you don't care to replace smoking with gum, what would need to happen for you to consider this change?']

['Although you don't want to quit smoking now and manage headaches in different ways, what type of changes would make you consider a change?']

['Although you are not dealing with quitting or hospitalization right now, what would need to happen for you to consider quitting smoking?']

['Even though you don't want to quit to improve hypertension now, what do you need in order to consider any type of change?']

['Although you are not dealing with ingredients and quitting smoking now, what would you need shifted in order to consider quitting?']

['Even though you don't desire to quit right now to improve your situation with inhalers, how could you be persuaded in doing things differently with smoking?']

['Although you don't need to quit to improve your lifestyle with liquor right now, what are some ways you can even think about changing smoking?']

['Even though you don't want to use lozenges for smoking cessation, what needs to happen for you to consider quitting?']

['Although you don't desire to quit smoking in order to improve your lungs, what would make you consider change?']

['What does this mean for you?']

['How could you benefit financially from quitting?']

['What other options do you have to help you with quitting?']

['How do you feel about the ingredients in a cigarette?']

['Which quit-smoking strategies might work better for you and why?']

['How would your parents feel if you quit?']

['If your cravings for niotine were taken away, would it make quitting a breeze?']

['What are your thoughts on cutting down before quitting entirely?']

['How could quitting smoking fit into your larger goals here?']

['How do you feel about laws that prevent smoking in public spaces?']

['What other things could motivate you to quit?']

['How could your sister support you in quitting?']

['How important is your appearance to you?']

['What are some things you don't like about smoking?']

['How might smoking be stigmatized in your community?']

['How does smoking get in the way of this?']

['Have you heard about how smoking can increase your risk of a stroke?']

['What other activities would improve if you quit?']

['How else could you benefit from quitting?']

['How could smoking affect your teeth?']

['What are some ways you can manage your triggers to smoke?']

['Are there other replacements for cigs?']

['For you, how does weed relate to tobacco use?']

['What alternative strategies could you use to manage weight concerns?']

['How does smoking affect your palate?']

['How might quitting help your hair and skin?']

['What other strategies could you rely on for stress-relief?']

**Affirmations**

Thank you for sharing that with me. I appreciate the opportunity to talk with you about your feelings.

Got it. Well, hopefully I can be helpful during our future chats. I get the sense that you can do whatever you set your mind to.

Great job showing up to explore obstacles, and putting thought into goals you want.

That's fascinating - thanks for being willing to consider different possibilities of choices you can make about your smoking.

From talking with you, I think you will give it your best effort when you're ready to quit .

Thanks for giving that some thought.

Thanks for joining today.
